# Impact of Managed Entry Agreements on availability of and timely access to medicines: an ex-post evaluation of agreements implemented for oncology therapies in four countries

**DOI:** 10.1186/s12913-022-08437-w

**Published:** 2022-08-20

**Authors:** Olina Efthymiadou, Panos Kanavos

**Affiliations:** Medical Technology Research Group, Department of Health Policy, London School of Economics, Houghton Street, London, WC2A 2AE England

**Keywords:** Risk sharing agreements, Managed entry agreements, Reimbursement, Access delays, Impact assessment

## Abstract

**Background:**

Despite the increased utilisation of Managed Entry Agreements (MEAs), empirical studies assessing their impact on achieving better access to medicines remains scarce. In this study we evaluated the role of MEAs on enhancing availability of and timely access to a sample of oncology medicines that had received at least one prior rejection from reimbursement.

**Methods:**

Funding decisions and their respective timelines for all oncology medicines approved between 2009 and 2018 in Australia, England, Scotland and Sweden were studied. A number of binary logit models captured the probability (Odds ratio (OR)) of a previous coverage rejection being reversed to positive after resubmission with vs. without a MEA. Gamma generalised linear models were used to understand if there is any association between time to final funding decision and the presence of MEA, among other decision-making variables, and if so, the strength and direction of this association (Beta coefficient (B)).

**Results:**

Of the 59 previously rejected medicine-indication pairs studied, 88.2% (*n* = 45) received a favourable decision after resubmission with MEA vs. 11.8% (*n* = 6) without. Average time from original submission to final funding decision was 404 (± 254) and 452 (± 364) days for submissions without vs. with MEA respectively. Resubmissions with a MEA had a higher likelihood of receiving a favourable funding decision compared to those without MEA (43.36 < OR < 202, *p* < 0.05), although approval specifically with an outcomes-based agreement was associated with an increase in the time to final funding decision (B = 0.89, *p* < 0.01). A statistically significant decrease in time to final funding decision was observed for resubmissions in Australia and Scotland compared to England and Sweden, and for resubmissions with a clinically relevant instead of a surrogate endpoint.

**Conclusions:**

MEAs can improve availability of medicines by increasing the likelihood of reimbursement for medicines that would have otherwise remained rejected from reimbursement due to their evidentiary uncertainties. Nevertheless, approval with a MEA can increase the time to final funding decision, while the true, added value for patients and healthcare systems of the interventions approved with MEAs in comparison to other available interventions remains unknown.

**Supplementary Information:**

The online version contains supplementary material available at 10.1186/s12913-022-08437-w.

## Background

The restricting cost containment environment in which healthcare systems are required to operate, introduces challenges on policy decisions about the coverage of highly priced pharmaceuticals. These challenges often arise as the evidence presented by manufacturers is not always sufficient to estimate the real-life budget impact, clinical and cost-effectiveness of these high-cost pharmaceuticals. More importantly, the uncertainties posed by the immature evidence submitted by manufacturers may prevent or delay healthcare payers from reaching conclusions on coverage decisions, thus affecting patient access [1].

Against this background, there is an interest from healthcare payers and manufacturers to collaboratively manage the entry of new pharmaceuticals in the market by linking price and reimbursement levels to real-world performance or utilization of medical products with the aim of sharing the risk surrounding the introduction of new technologies with uncertain evidence on their clinical and/or cost-effectiveness profiles. Prices can be linked to future outcomes and/or volumes and the specific conditions of the negotiations are drawn up into product listing agreements usually summarised as Risk Sharing Agreements (RSAs), Managed Entry Agreements (MEAs) or Patient Access Schemes (PAS) [2–4]. The main types of these agreements are financial-based and health outcomes-based agreements, or occasionally combination of both types. The former includes agreements at the population level (e.g., simple discounts or price–volume agreements) or at the patient level (e.g., utilisation, time, or cost capping schemes), and the latter includes performance-linked schemes (e.g., conditional treatment continuation, outcome guarantee and coverage with evidence development) [5].

It has been suggested that MEAs can improve access to innovative medicines by addressing decision-making related uncertainties and hence, preventing rejection from reimbursement due to uncertain clinical and cost-effectiveness evidence [6–8]. Nevertheless, these agreements have not yet gained widespread acceptance primarily because their sustainability is unclear and their effectiveness in meeting their objectives has yet to be evaluated [9]. Key issues around the efficiency of MEAs relate to the often lengthy or stalled MEA negotiations causing access delays, and the risk for a product reimbursed with a MEA being delisted following expiry of the agreement thus, impeding patient access [5]. Another area of concern in the implementation of MEAs relates to the administrative burden they are often associated with [7], especially for agreements that require advanced infrastructure systems to support new data generation [10].

Despite the significant attention placed on the implementation of MEAs, the body of evidence on the performance of MEAs to date is weak, as there is still little information on their real-life impact on patients and healthcare systems [11, 12]. The main body of literature attempting to evaluate MEAs is based on theoretical models that assess the economic impact of MEAs [13–18]. Additionally, the role of MEAs in achieving a meaningful impact on key policy objectives such as cost containment, improved access and reward of innovation, has been discussed in the literature chiefly in the context of describing their “strengths and weaknesses” [3, 7, 19]. The key challenge in conducting empirical impact assessments for MEAs arises due to the confidentiality and limited information available on the specific negotiating terms and operational details of these agreements (i.e., timeframe, patient eligibility, indicators used to monitor outcomes etc.) [3, 11]. Only a few empirical studies exist on the real-life impact of implemented MEAs on pharmaceutical expenditure [20, 21], list prices [11], faster access to cancer medicines [22] and on the ability of outcomes-based schemes to collect meaningful, long-term outcomes data for patients [23, 24]. Additionally, existing empirical literature primarily reflects case studies within one specific setting/country and hence, comprehensive evidence about the broader effectiveness of MEAs in meeting their anticipated objectives remains scarce [9, 25, 26]. For example, Russo et al., (2010) [22] assessed the impact of MEAs on access delays only from the Italian healthcare system perspective and concluded that the impact of MEAs remains equivocal due to diverse health system priorities, different assessment criteria, different market access/purchasing strategies and market sizes across different countries. Other studies concluded that despite MEAs’ potential to improve access, there is no consensus on which MEA types and implementation strategies are the most effective in optimising reimbursement decision-making [13].

Drawing more robust conclusions about the pragmatic impact of MEAs is paramount to understand if these agreements represent a sustainable policy tool for improved coverage across countries. This could also help purchasers to identify the most efficient MEA negotiation practices by understanding which situations call for the use of one type of MEA instead of another, and what trade-offs are involved in choosing different contracts [13]. To that end, structured ex-post evaluations of MEAs are essential to assess the impact of existing schemes on a number of key policy goals such as access to medicines, budget control and encouragement of innovation [4, 8, 27]. In practice, these evaluations can take the form of quantitative models that enable the outcomes of these agreements to be compared with those in situations without them [9, 11].

We are not aware of any other empirical studies that involve direct comparisons of MEAs to understand how these agreements influence the level of and/or speed of access to medicines across countries. Therefore, the objective of this study was to contribute evidence around the impact that completed agreements or resubmissions with an agreement have had on a) the levels of access (*i.e.*, resulting in more “listing” recommendations) and b) the time taken to the final decision outcome. These objectives were selected for impact assessment because first, they reflect a key policy goal targeted by health systems across borders [28] and second, because of relevant data availability that ensures feasibility of the required data analysis.

## Methods

### Sample selection

This study was based on a retrospective analysis of HTA appraisals for all oncology medicines which obtained regulatory approval by the European Medicines Agency (EMA) in Europe and by the Therapeutic Goods Administration (TGA) in Australia between 1^st^ January 2009 and 15^th^ June 2018 (at the medicine-indication pair level) in Australia, England, Scotland and Sweden. Oncology was the therapeutic area of choice because it has been documented to be the therapeutic class with the largest proportion of implemented MEAs, while also being the therapeutic class where MEAs continue to be increasingly implemented [3].

Study countries were selected because they all implement MEAs, they all have long-established HTA policies and processes to guide their coverage decisions/ recommendations, they have both a publicly available list of MEAs and publicly available HTA reports which provide sufficient information for the purposes of this analysis, [29]. Additionally, these countries were selected because, apart from the cost-effectiveness perspective, they also use other, different principles to shape their decision-making around pricing and reimbursement of medicines (e.g., England also considers the national health and personal social services perspective and Sweden also takes into account the human value and solidarity principle (further information about the study HTA agencies and their respective HTA perspective is provided in supplementary material; see Appendix Table 1). Therefore, countries were selected such that they would allow for comparability across agencies, while reflecting the diversity in HTA coverage decisions/recommendations and the respective HTA determinants of access across settings [29].

**Table 1:** Binary logit models, predicting the likelihood/ odds ratio (OR) of a previously negative coverage decision being reversed to a favourable funding decision, based on the set of HTA predictors studied in the model

	**Model 1**		**Model 2**		**Model 3**		**Model 4**^**a**^		**Model 5**	
**HTA Predictor**	*OR*	*p*	*OR*	*p*	*OR*	*p*	*OR*	*p*	*OR*	*p*
HTA agency		.916		1.0						.180
**MEA in place**	**43.36**	**.017**	**63.35**	**.012**	**202**	**.008**	**.005**	**.004**		
Orphan designation	62.56	.091	91.29	.108	108.5	.178	.004	.065		
Year MA					1.65	.337				
**Endpoint**										
**Surrogate**	.024	.063	**.017**	**.030**	**.019**	**.042**	.113	.289	.000	.997
**Clinical**	.001	.124	.007	.066	.005	.054	**50.96**	**.037**	.000	.997
Study type					2.08	.798	.490	.800		
Uncertainties										
Clinical evidence			2.734	.505	5.67	.367	.403	.567	3.04	385
**Clinical benefit**	.094	.206	.065	.132	**.021**	**.044**	**53.60**	**.024**	.000	.997
Utilities	.022	.251								
Cost effectiveness									.000	1.0
Social Value Judgements										
Special considerations			.000	.999						
Severity	.731	.905							.477	.705
Unmet need					2.58	.563	.658	.774	.341	.388
Administration advantage	16.39	.998								
Constant	.000	.998	.195	.713	.000	.335	.632	.772	61.4	.999
**Model statistics**	*χ*^2^	*p*	*χ*^2^	*p*	*χ*^2^	*p*	*χ*^2^	*p*	*χ*^2^	*p*
Likelihood ratio test	31.15	.002	30.84	.001	25.67	.004	28.73	.001	46.53	.000
Hosmer & Lemeshow test^b^	1.76	.972	1.76	.971	5.11	.646	5.76	.568	5.10	.647
Predictability (%)	94.8%		94.8%		94.6%		94.8%		82%	
Nagelkerke R^2^	75.3%		74.8%		69.5%		70.8%		54.6%	

^a^Categorical HTA predictors were treated as binary variables taking the outcomes 0 = not raised/not considered/not in place vs. 1 = raised/considered/in place (and 0 = Surrogate vs. 1 = Clinical for the “Endpoint” variable); the second outcome of each HTA predictor was used as a reference category for all models, apart from model 4 where the first outcome was used

^b^The Hosmer–Lemeshow test has been used as a goodness of fit test to indicate how well the data fits each model; it is not provided as a comparison or grading metric between the different competing models, neither it has been used for selecting the best model

*HTA* Heath Technology Assessment, *MA* Marketing authorization, *MEA* Managed Entry Agreement, *OR* Odds Ratio, *p*: *p*-value

### Variables of interest

From the sample described above, all medicine-indication pairs with a resubmission following an HTA rejection and all medicine-indication pairs with a resubmission following completion/expiry of a previously agreed MEA identified and isolated separately for analysis; none of the respective MEAs were implemented across multiple indications of a specific molecule and/or were part of a Multi-Year Multi-Indication (MYMI) agreement. Further information about the medicine-indication pairs studied is provided in supplementary material (see Appendix Table 2). Among these medicine-indication pairs, three main categories of variables were collected and studied for the purposes of this study. These included:

1. *Previous and final funding decision outcome (i.e.,* prior to and following a resubmission with and without a MEA) classified as (i) favourable recommendation/ decision, including “List” (L) without restrictions/criteria, “List with criteria” (LWC) and “LWC with MEA as part of the listing criteria” (LWCMEA), and (ii) non-favourable or “do not list” (DNL) HTA funding recommendation/decision.
2. *HTA decision-making determinants,* based on a conceptual framework described elsewhere [29, 30] dividing the HTA appraisal and assessment processes in three main stages and respective variables therein, corresponding to (i) the evidence submitted (*e.g.,* trial characteristics and endpoints used, size of clinical benefit and existence or not of a MEA), (ii) the interpretation of this evidence (*i.e.,* clinical and economic evidence related uncertainties raised), and (iii) Social Value Judgements (SVJs) and system-specific considerations (*i.e.,* dimensions of value that a technology adds, beyond its clinical evidence/benefit and cost-effectiveness such as innovation, the severity, rarity and unmet need of the targeted disease or process specific characteristics, as well as type of HTA system.
3. *Time* from previous submission to resubmission with vs. without MEA and to final decision outcome.

**Table 2:** Generalised linear models, predicting the association between a set of HTA predictors and time to final reimbursement decision

	**Model 1**		**Model 2**			**Model 3**		**Model 4**	
**HTA Predictor**	B	*p*		B	*p*	B	*p*	B	*P*
**HTA agency**^**a**^		***.000***			***.007***				.***000***
NICE	-.030	*.926*		.245	*.528*			-.018	*.955*
**PBAC**	**-1.019**	***.001***		-.709	*.053*			**-.986**	***.001***
**SMC**	**-.815**	***.003***		-.453	*.226*			**-.790**	***.004***
MEA in place	-.179	*.416*							
Type of MEA					*.453*		***.000***		
Financial				-.100	*.706*	-.145	*.569*		
**Outcomes-based**				.379	*.299*	**.897**	***.008***		
Orphan designation	.088	*.618*				.287	*.132*		
Endpoint		*.054*			*.146*		*.756*		*.085*
Surrogate	-.301	*.205*		-.340	*.132*	-.057	*.815*	-.324	*.143*
**Clinical**	**-.612**	***.022***		**-.562**	***.034***	-.098	*.690*	**-.559**	***.033***
Uncertainties									
**Study design**	-.262	*.118*		-.285	*.089*	**-.361**	***.019***	**-.329**	***.043***
**Clinical evidence**	.247	*.114*		**.336**	***.038***	**.482**	***.002***	.221	*.150*
Clinical benefit	-.212	*.238*		-.189	*.288*				
Cost effectiveness	.050	*.934*		.052	*.929*			.041	*.943*
Social Value Judgements									
Severity	.176	*.378*		.136	*.502*	-.221	*.173*	.136	*.490*
**Societal impact**	**.623**	***.005***		**.690**	***.002***	**.628**	***.007***	**.603**	***.004***
Constant	17.13	*.841*		-28.67	*.735*	5.62	*.000*	3.54	*.965*
**Model statistics**	*χ*^2^	*p*		*χ*^2^	*p*	*χ*^2^	*p*	*χ*^2^	*p*
Likelihood ratio test	45.30	*.000*		47.01	*.000*	34.70	*.000*	43.43	.000
Deviance (Value/df)	.407			.406		.430		.395	

^a^The “HTA agency” was treated as a multinomial variable taking the outcomes 0 = NICE, 1 = PBAC, 2 = SMC, 3 = TLV, whereby the last outcome (i.e., TLV) was used as a reference category for all models. All other categorical HTA predictors were treated as binary variables taking the outcomes 0 = not raised/not considered/not in place vs. 1 = raised/considered/in place (and 0 = Surrogate vs. 1 = Clinical for the “Endpoint” variable); the second outcome of each HTA predictor was used as a reference category for all models

*B* Regression coefficient, *df* Degrees of freedom, *HTA,* Heath Technology Assessment, *MEA* Managed Entry Agreement, *p*: *p*-value, PBAC: Pharmaceutical Benefits Advisory Committee, *NICE* National Institute for Health and Care Excellence, *SMC* Scottish Medicines Consortium, *TLV* Dental and Pharmaceutical Benefits Board

Data on the above variables per medicine-indication pair in all study countries were extracted only from the official, publicly available HTA appraisals, which were published in the websites of the respective HTA bodies, namely the Pharmaceutical Benefits Advisory Committee (PBAC) in Australia, the National Institute for Health and Care Excellence (NICE) in England, the Scottish Medicines Consortium (SMC) in Scotland and the Dental and Pharmaceutical Benefits Board (TLV) in Sweden. Other relevant sources of data, such as the county councils' group on new drug therapies in Sweden were not searched. Data collection was undertaken between June and December 2018 and data extracted was put in a database stratified by HTA agency.

### Data analysis

Funding decision outcome was coded as a binary variable (*e.g.,* positive and negative reimbursement decision), uncertainties and SVJs were coded as binary variables based on whether they have been raised and considered (or not) respectively in the decision-making process, and variables around the evidence submitted were treated as binary (*i.e.,* existence of MEA or not), continuous (*i.e.,* time to final funding decision) or categorical (*i.e.,* type of MEA, type of endpoint etc.) depending on their specification.

For the first part of the analysis Pearson's chi-squared and where applicable, *t-*tests were performed for all HTA decision-making determinants, and the variables driving significant differences between positive and negative funding decision outcomes, were selected for further analysis. Subsequently, we examined the probability of a previously negative funding decision being reversed to positive following a resubmission, based on the key HTA variables of significance identified, including existence/non-existence of MEA (as a proxy for the impact of MEAs on enhancing availability of medicines). As the dependent variable for the first part of the analysis is categorical, a non-linear, cumulative logit model was chosen, namely a binary logit model, to model the probability (*P*) of a previously rejected technology receiving a favourable funding decision after resubmission (*y*_*i*_ = 1) (as opposed to remaining rejected), based on a set of explanatory variables (*x*_*i*_), under the following Eq. (1):

1 $$P\left({y}_{i}=1 \right| {x}_{i})= \frac{\mathit{exp}({x}_{i}\beta )}{1+\mathit{exp}({x}_{i}\beta )}$$

where:

- *y* is a binary response variable with:
  - *y*_*i*_ = 1 if the resubmission resulted in a positive funding decision
  - *y*_*i*_ = 0 if the resubmission resulted in a negative funding decision
- *x* = *(x*_*1*_*, x*_*2*_*, …, x*_*k*_) is a set of HTA explanatory variables hypothesised to influence HTA decision-making, and a distinct explanatory variable on presence of a MEA (or not) as part of the resubmission whereby:
  - *x*_*i*_ is the observed value/outcome of the respective explanatory variables tested and
  - *β* is a vector of parameters to be estimated and presented as Odds Ratio (OR) (*e.g.,* a one-unit change in the *j*th variable, *x*_*j*_, is associated with the OR, *exp*(*β*_*j*_) [31].

For the second part of the analysis we captured the relationship between the time to final funding decision and existence of a MEA (including both resubmissions with MEA following a previously negative funding decision and resubmissions following expiry of a MEA), as a proxy for the impact of MEAs on market access delays. First, Mann–Whitney U and Kruskal–Wallis (where applicable) tests were performed to assess if there is a statistically significant association between any of the HTA predictors (including presence of a MEA or not) and the average time to final funding decision. Subsequently, given the non-normally distributed, exponential (*i.e., gamma)* distribution of the average time to final funding decision, a gamma generalised linear model with log link function was performed to identify the strength and direction of the above association. This model was employed as the best fit of a regression model for a non-Gaussian distribution, and is described by the following Eq. (2):

2 $$g\left({\mu }_{i}\right)={\varvec{X}}{}_{i}{}^{\mathbf{T}}{\varvec{\beta}}= {\beta }_{0 }+ \sum_{j=1}^{P}{x}_{ij}{\beta }_{j}$$

where:

- ${\mu }_{i}= {\mathbb{E}}({Y}_{i})$ is the expected value of the response Y_*i*_ given the predictors
- *g*(⋅) is a smooth and monotonic link function that connects *μ*_*i*_ to the predictors
- ${\mathbf{X}}_{i}^{T}=$ (*x*_*i0*_*, x*_*i1*_,..., *x*_*ip*_) is the *i*-th observation’s known predictor vector with X_*i0*_ = 1 and
- ***β*** = (*β*_*0*_*,β*_*1*_*,...,βp*)^T^ is the unknown vector of regression coefficients.

A log-link function was applied in the above to exponentiate the linear predictors as follows:

$$\mathit{ln}\left(\mu \right)= {\beta }_{0}+ {\beta }_{1}X \Rightarrow \mu = \mathrm{exp }(\beta 0 + \beta 1{\rm X}) ,$$

where *μ* is the predicted value of *Y* given X, exp(*β*_*0*_) is the effect on the mean of *μ* when X = 0.

and exp(*β*_*1*_) is the multiplicative effect on the mean of *Y* for a one-unit increase in X.

The SPSS® (v.24.0) was used to perform the econometric models and statistical tests, and Excel® 2013 to generate descriptive statistics, where relevant.

## Results

### Impact of MEAs on reimbursement decisions

#### Descriptive statistics

Of the 59 resubmissions studied, 1.7% (*n* = 1) were reversed to L, 8.5% (*n* = 5) were reversed to LWC, 76.3% (*n* = 45) reversed to LWCMEA, and 13.5% (*n* = 8) remained rejected. Overall, of the 59 previously rejected medicine-indication pairs 86.5% (*n* = 51) received a positive reimbursement decision after resubmission and of these, 88.2% (*n* = 45) achieved so with a MEA vs. 11.8% (*n* = 6) without (see Appendix Table 3). Furthermore, *χ*^2^ tests were also performed to assess if there is any statistically significant association between any of the HTA predictors and/or molecule specific characteristics and the final funding decision following a resubmission. It was demonstrated that a statistically significant difference between positive and negative decisions following resubmission is underscored by the existence or not of a MEA (*p* < 0.001) and existence or not of cost effectiveness uncertainties (*p* < 0.05) (see Appendix Table 3). All descriptive statistics on the final funding decision outcomes after resubmission and statistical significance (*p*) of their HTA determinants are provided in supplementary material (see Appendix Table 3).

#### Binary logit model

According to the *χ*^2^ tests presented above, only the existence or not of a MEA (*p* < 0.001) and existence or not of cost effectiveness uncertainties (*p* < 0.05) were shown to play a role in determining the funding decision outcome following resubmission of evidence for a previously rejected medicine-indication pair. A number of binary logit models were performed to ascertain the effects of the above variables, in consideration with a combination of other HTA predictors, on determining the likelihood of a previously non-favourable coverage decision being reversed to favourable.^1^ The models with the best predictability rate are presented below (Table 1).

The first model was statistically significant (*χ*^2^ = 30.84, *p* = 0.002), it explained 75.3% (Nagelkerke R^2^) of the variance in the funding decision outcomes and correctly classified 94.8% of cases. In this model, a resubmission with a MEA was the only positive predictor of receiving a favourable funding decision instead of non-favourable (OR = 43.36, *p* = 0.017). Other HTA parameters included in the model did not have a statistically significant effect in the overall model.

The second model was statistically significant (*χ*^2^ = 30.84, *p* = 0.001), it explained 74.8% (Nagelkerke R^2^) of the variance in the funding decision outcomes and correctly classified 94.8% of cases. Resubmission with a MEA was the only positive predictor of a previously negative coverage decision being reversed to positive, although the positive effect was stronger (OR = 63.35, *p* = 0.012) compared to the previous model. Additionally, resubmission with a surrogate endpoint was a negative predictor (OR = 0.017, *p* = 0.03) of a previous rejection being reversed to a favourable funding decision.

The third model was statistically significant (*χ*^2^ = 25.7, *p* = 0.004), it explained 69.5% (Nagelkerke R^2^) of the variance in the funding decision outcomes and correctly classified 94.6% of cases. Resubmission with a MEA was the only positive predictor of a previously negative coverage decision being reversed to positive, and the positive effect was the strongest (OR = 202, *p* = 0.007) compared to the previous models. Additionally, in this model there were two negative predictors in achieving a positive reimbursement decision, namely the use of a surrogate instead of clinical outcome and the presence of clinical benefit uncertainties in the resubmitted evidence, with the former being a slightly stronger negative predictor (OR = 0.019, *p* = 0.042) compared to the latter (OR = 0.021, *p* = 0.044).

The fourth model was statistically significant (*χ*^2^ = 28.73, *p* = 0.001), it explained 70.8% (Nagelkerke R^2^) of the variance in the funding decision outcomes and correctly classified 94.8% of cases. In this model, a resubmission without a MEA was a negative predictor (OR = 0.005, *p* = 0.004) of a non-favourable decision being reversed to favourable. Additionally, resubmission without clinical benefit uncertainties in the evidence submitted was the strongest positive predictor (OR = 53.608, *p* = 0.024) of a previously non-favourable decision being reversed to favourable, followed by resubmission with a clinically relevant endpoint (OR = 50.965, *p* = 0.037) as opposed to a surrogate.

Finally, since the presence of cost-effectiveness uncertainties seemed to drive a statistically significant difference between a favourable and non-favourable funding decision outcome following a resubmission (see Appendix Table 3), a number of models were also performed to ascertain the effect of the “cost effectiveness uncertainties” variable on reversing previously negative decisions. Only one model (Model 5; Table 1) was found to be of statistical significance (*χ*^2^ = 46.538, *p* < 0.001) but this had a relatively poor predictability and variance explanation (Nagelkerke R^2^) rates (82% and 54.6% respectively), compared to the models presented above. Moreover, none of the predictors included in this model, including the “cost-effectiveness uncertainties” variable contributed a statistically significant effect in the model.

### Impact of MEAs on time to reimbursement decisions

#### Descriptive statistics

**Fig. 1:**
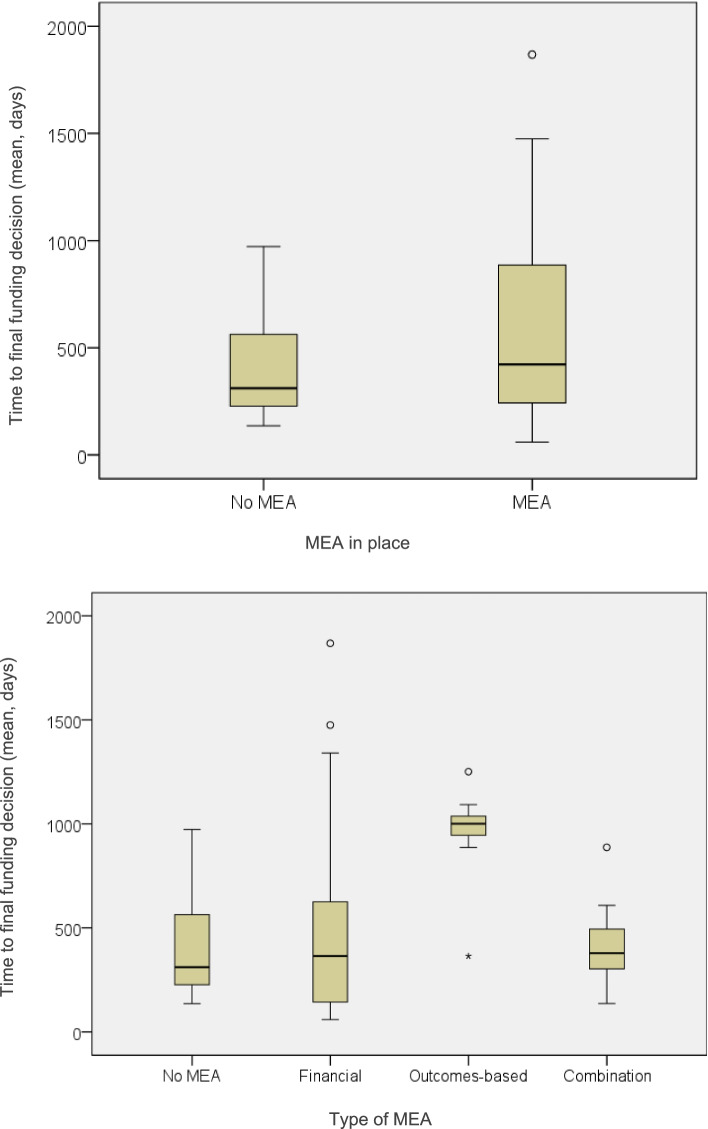
Average time from initial to final funding decision following resubmission without vs. with MEA, and the respective time exhibited by resubmissions with different MEA types. Key: Time represents average days from first submission to final funding decision after resubmission; Horizontal lines indicate medians; Boxes indicate interquartile range; Single points indicate outliers

Medicine-indication pairs with a resubmission following a previously negative funding decision and those with a resubmission/re-evaluation following MEA expiry were studied. Across the 71 re-submissions and re-evaluations studied, 83% (*n* = 59) were resubmissions following a previous rejection and 17% (*n* = 12) were resubmissions/re-evaluations after expiry of a MEA. Average time to final funding decision across all sample was 525 (± 386) days, and this was 452 (± 364) and 404 (± 254) days for medicine-indication pairs approved with vs. without a MEA respectively (Fig. 1; Appendix Table 4).

**Fig. 2:**
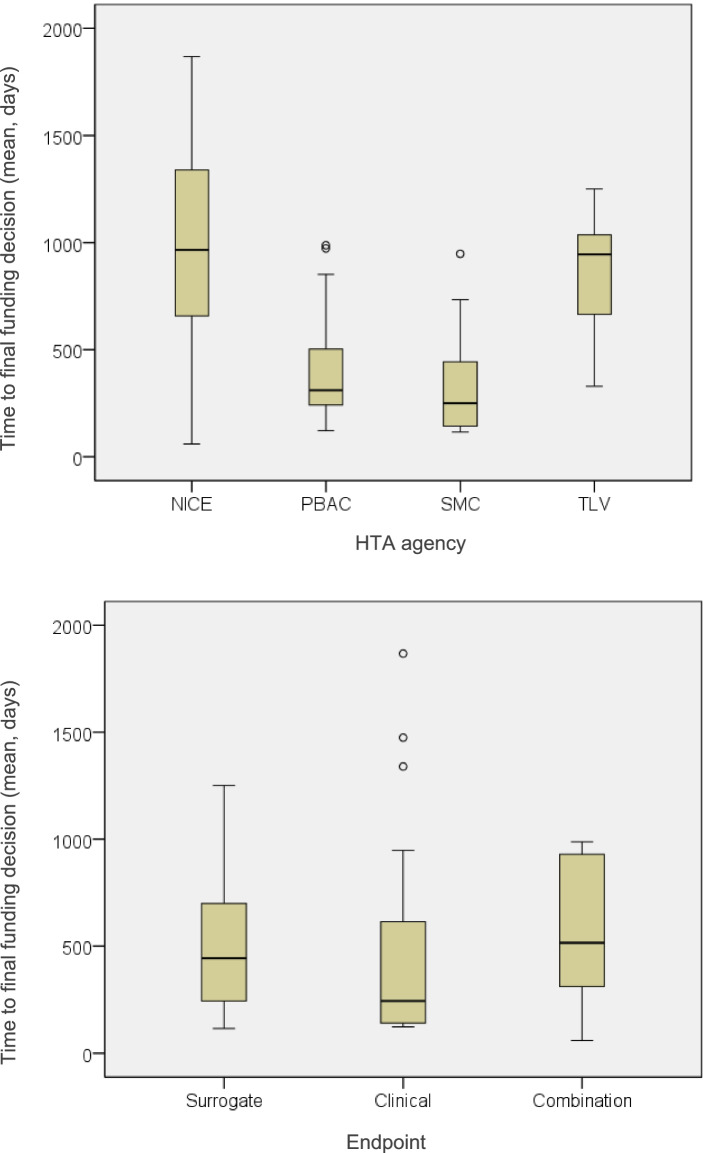
Average time from initial to final funding decision after a resubmission, between the different HTA agencies and types of endpoints. Key: Time represents average days from first submission to final funding decision after resubmission; Horizontal lines indicate medians; Boxes indicate interquartile range; Single points indicate outliers. Note: PBAC: Pharmaceutical Benefits Advisory Committee (Australia), NICE: National Institute for Health and Care Excellence (England), SMC: Scottish Medicines Consortium (Scotland), TLV: Dental and Pharmaceutical Benefits Board (Sweden)

The Mann–Whitney U and Kruskal–Wallis tests demonstrated that a statistically significant difference in mean time to final funding decision was underscored by the type of HTA agency (*χ*^2^ = 23.587, *p* < 0.001), type of MEA (*χ*^2^ = 14.634, *p* = 0.002) and the SVJs of disease severity (*U* = 342.5, *p* = *0.013)* and societal impact (*U* = 159.5, *p* = 0.044). Among the above predictors, the greatest differences in average time to final funding decision existed between the different types of MEAs and different HTA agencies (see Appendix Table 4). More precisely, in terms of differences underpinned by the different MEA types, it was shown that shortest mean time to final funding decision was 422 (± 231) days for medicine-indication pairs with a combination of a financial and outcomes-based schemes, followed by 476 (± 407) days for medicine-indication pairs with a financial agreement and amounting up to 957 (± 231) days for medicine-indication pairs approved with an outcomes-based agreement (Fig. 1). Finally, in terms of time differences between HTA agencies, the shortest mean time to final funding decision was 342 (± 249) days for the Scottish HTA agency, followed by 378 (± 242) days for the Australian agency, 837 (± 302) days for the Swedish agency and reaching an average of 938 (± 559) days for the English agency (Fig. 2). All descriptive statistics on the time (days) elapsed from initial to final funding decision after resubmission, and statistical significance (*p*) of their HTA determinants are provided in supplementary material (see Appendix Table 4).

#### Generalised linear models

Gamma generalised linear models were performed to ascertain the effects of several HTA predictors on the average time taken to reach a final funding decision (Table 2).

In the first model, variables with a statistically significant impact on time to final funding decision were HTA agency (*p* < 0.001), the use of clinical endpoint in the evidence submitted (*p* = 0.022) and the SVJ of societal impact (*p* = 0.005). The Australian and Scottish agencies were associated with a reduction in time to final funding decision, as was the use of a clinically relevant endpoint in the evidence submitted. Absence of considerations around the societal impact of the technology in question increased the time to final funding decision, whereas the presence of a MEA did not have a statistically significant contribution in the overall model.

The second model examined the impact of HTA agency and the type of MEA on the average time to final funding decision. Variables with a statistically significant contribution in the model were HTA agency (*p* = 0.007), the type of endpoint used in the clinical evidence submitted (*p* = 0.034), clinical evidence uncertainties (*p* = 0.038) and the SVJ of societal impact of the technology in question (*p* = 0.002). Submissions with a clinically relevant endpoint were associated with a reduction in time to decision-making. Raising considerations around the societal impact of the technology in question and raising uncertainties around the clinical evidence submitted had a positive impact (*i.e.*, increase) on time to final funding decision. Finally, the type of MEA did not have a statistically significant contribution in the overall model.

Controlling for HTA agency, the third model examined the role of the type of MEA on time to final decision. Variables with a statistically significant contribution in the model were the type of MEA (*p* < 0.001), the HTA agency (*p* = 0.007), uncertainties around the study design (*p* = 0.019) and the clinical evidence submitted (*p* = 0.038), and the SVJ of the societal impact of the technology (*p* = 0.002). Submissions with an outcomes-based agreement increased the time to decision-making. Raising considerations around the societal impact of the technology and raising uncertainties around the clinical evidence submitted increased the time to final funding decision, whereas presence of study design uncertainties had a negative impact (*i.e.,* decrease) on time to final funding decision.

## Discussion

We conducted an analysis of oncology medicines previously rejected from reimbursement, to understand if any MEAs implemented upon evidence resubmission of the above medicines had an impact on enhancing the availability of and timely access to these medicines. Our results suggest that presence of MEAs has the potential to improve the availability of new oncology therapies, by increasing their likelihood for reimbursement if they have previously been rejected. However, presence specifically of outcomes-based agreements can cause significant time delays in reimbursement decision-making and hence, time to access.

Only a few studies have provided a quantitative evaluation of the impact of MEAs on access to medicines [12, 22, 32–34]. In Italy, it was shown that the introduction of MEAs contributed substantially to an improvement in patients’ access to cancer medicines [12, 34], whereas in Finland and South Korea it was estimated that about 20% and 60% of patented medicines respectively were granted reimbursement due to the presence of a MEA, and of the 60% reimbursed in the later, 23% were previously rejected [32, 33]. Similarly, in Australia, MEAs have been implemented as part of the government’s plan to enhance access to medicines, estimating that MEA implementation can help achieve coverage for about one-third of new medicine-indication pairs [35].

It has also been suggested that reimbursement with a MEA, regardless of its type, can improve time to patient access [22, 36]. We found that, medicine-indication pairs approved with a MEA exhibited longer average time to final reimbursement decision, although only the presence of an outcomes-based agreement specifically (as opposed to presence of a MEA in general) was associated with a statistically significant increase of about 480 days to final funding decision. Comparable findings have been reported by a study of oncology medicines in the Italian setting, which showed an increase in the national time to market of about 150 days for medicines approved with an outcomes-based agreement compared to those approved with a financial scheme [34]. This finding is not surprising; the complexity of outcomes-based contracts in comparison to more simple financial schemes, their negotiation process can often be burdensome and time consuming for manufacturers and payers. Additionally, the collection of additional evidence and if required, the future monitoring and re-assessment of the product, as well as the need to align interpretations of the collected and required data between the different stakeholders involved in reimbursement decision-making may introduce further delays [10, 37, 38].

Discrepancies in the conclusions of existing literature around the impact of MEAs on time to access may be explained on the grounds that regardless of their type, MEAs can only improve time to market access if negotiation processes are well structured and based on sufficient preparation ahead of time such that the proposed schemes have a clear rationale and truly address the uncertainties raised by the competent authorities assessing the technology in question [39]. Growing concerns have been expressed in the literature that MEAs are increasingly used as “an operational tool” to agree on commercial price negotiations and confidential discounts rather than as a tool for managing the actual risk arising from immature data [40]. Therefore, even simple financial schemes need to be implemented such that they meaningfully address the uncertainties that a new therapy presents with, rather than implemented simply as a tool to achieve lower prices. More importantly, when financial schemes are used solely as a cost containment process on top of other cost containment policies, they can add little benefit in terms of outcomes for patients and increase delays in the long term [41]; for example, they might grant access to interventions which might prove cost-ineffective in the long-run with the consequence that these technologies will be delisted after expiry of the agreement and eventually harm patient access, if there is no comprehensive risk management plan in place, in case of delisting [42].

The findings arising from this study suggest that presence of a MEA per se may not always guarantee a favourable funding decision and/or faster access to oncology medicines. There are additional HTA decision-making variables which determine the final reimbursement decision and the time taken to final decision. More precisely, this study highlights that successful and timely access to oncology therapies is also subject to submission of clinical evidence which presents with minimal uncertainties and is primarily based on clinically relevant instead of surrogate endpoints. Literature has also underscored the importance that HTA decision-makers place on submitting evidence with clinically meaningful outcomes relating to mortality, morbidity, and quality of life [43]. Even though the use of surrogate measures in cancer medicines’ trials is not associated with an HTA decision to reject a medicine [44], a gap between the surrogate endpoint and the final clinical endpoint creates additional uncertainty for decision-makers. Consequently, in this case, decision-makers often need to engage in additional validation processes to extrapolate findings beyond the submitted evidence to estimate the expected true benefits for patients and health systems, and this translates in further delays on the time required to reach a final reimbursement decision [45, 46].

Additionally, it was demonstrated that uncertainties around the study design had a statistically significant contribution in the model explaining time to final reimbursement decision. This was not surprising given that the trial design is often taken into consideration by some HTA agencies, such as SMC where for example, an active-controlled trial is preferred over a placebo one [47]. In the generalised linear model, the “study design uncertainties” variable was negatively associated with time, potentially demonstrating that this specific type of clinical uncertainty might lead to a confident, outright rejection and thus, shorten time to decision-making. This is in alignment with the results presented elsewhere [30], demonstrating that the presence of clinically relevant uncertainties is not typically associated with the flexibility to enter into negotiations for restricted reimbursement.

Finally, it was demonstrated that time to final funding decision can also be influenced by the HTA agency involved in the decision-making process. In our study, the Australian and Scottish HTA agencies exhibited significantly shorter timelines to final funding decision compared to the Swedish and English agencies. Comparable findings have been reported elsewhere. For example, a study assessing the delays introduced by HTA processes across countries in their coverage decisions for oncology medicines, showed that in England median time from EMA regulatory approval date to NICE decision was 783 days, as opposed to an average of 231 days required for SMC decisions [48]. Similarly, more recent figures estimated the mean length of time from EMA authorization to HTA funding decision for oncology and all products at 436 and 335 days respectively for NICE, compared to for example 389 and 262 days respectively for TLV [49]. Overall, it has been reported that NICE exhibits relatively higher timelines to final funding decision compared to other European HTA agencies [49]. On the contrary, as demonstrated in this study, Australia has been reported to have the fastest median timelines from TGA approval to HTA recommendation at national level (127 days) compared to other jurisdictions, including England (386 days), Scotland (293 days) and Sweden (217 days) [50].

Relevant literature suggests that these differences in time to decision-making are shaped by agency specific characteristics and procedures. Specifically for oncology medicines, evidence demonstrates that divergent HTA methodologies across countries underline differences in the time required for new products to enter the market when considering the average time between date of regulatory approval and date of funding decision [51]. For example, since 2011, the TGA/PBAC parallel process has been introduced in Australia and this played an important role in streamlining the regulatory and reimbursement processes, leading to a significantly shortened time gap between marketing authorisation and first funding decision [50, 52]. On the contrary, in England, delays may often occur due to NICE specific modalities such as switching to the Cancer Drugs Fund during the review process [53]. Additionally, in England, time delays due to NICE procedures related specifically to MEA implementation processes have been reported. For example, the PAS Liaison Unit (PASLU) process may delay submissions to NICE, whereby specifically for Single Technology Appraisals the existence of a PAS can result in an average time delay of up to four months compared to Multiple Technology Appraisals with a PAS [53, 54]. In other markets, there is greater flexibly in the negotiation of these agreements with the result that this can eventually accelerate the decision-making process [55], such as in Italy where presence of an agreement typically leads to shorter time to patient access [12, 22]. The above further highlights that time delays associated with the presence of MEAs can be attributed to agency specific procedures for the implementation and negotiation of MEAs [56].

This is the first study to date to conduct a post-implementation evaluation of MEAs across countries, to quantify their impact on two key healthcare system policy goals, namely availability of and timely access to medicines. Since the on-going literature debate on the weaknesses of MEAs is primarily generated by the poor and inconclusive evidence as to whether these agreements have managed to meet their objectives, this study addresses important literature gaps on structured, impact assessment studies of MEAs. More importantly, the conclusions arising from this study can facilitate future policy relevant research around the sustainability of MEAs as an effective funding modality that can be applied for greater and faster access to medicines. Another strength of this study is the holistic approach taken in studying the HTA factors that determine coverage decision outcomes and timelines, whereby we accounted for the role of MEAs as well as the interconnected impact of both uncertainties, SVJs and clinical evidence characteristics, as opposed to existing literature that studies the impact of evidentiary uncertainties or MEAs individually.

Our study is not without limitations. First, accuracy of the models performed would have benefited from a larger sample size; although this study provides a good basis for future analyses, it is recommended that replication of similar analyses in the future could increase the sample size, possibly by including assessments of medicines for other therapeutic areas.

Second, we recognize that the cost-effectiveness and “added value” profile of the studied medicine-indication pairs is not equivalent within and across countries and hence, the need to apply a MEA would not always be equally applicable for all medicine-indication pairs studied. To address the limitation of having an unbalanced panel as our study sample, the impact of MEAs on promoting availability was studied only on medicine-indication pairs that were previously rejected, such that a common selection criterion (*i.e.,* previously cost-ineffective profile) would be established for all medicine-indication pairs in the analysis.

Third, accounting for the reversibility of negative to positive funding decisions as a proxy to availability of medicines is an assumption made for the purposes of simplicity in running the binary logit model. This assumption is a potential limitation of the analysis, since a positive reimbursement decision does not always translate in equal availability of the respective medicine; beyond a favourable funding decision other, macro-economic, country specific and healthcare system specific factors determine the actual availability of and patient access to medicines [55]. Similarly, accounting for the time to final funding decision as a proxy to timely access to medicines was an assumption made for simplicity in running the generalised linear model. This is also a potential limitation of our study, given that (as described above) a positive reimbursement decision does not always reflect ready access to the respective medicine, regardless of how promptly the funding decisions might have been reached. Finally, in the above context, it is also important to recognise that binding HTA outcomes (e.g., Sweden) typically correspond to funding decisions, whereas non-binding HTA outcomes (e.g., England, Scotland, Australia) correspond to recommendations, which are not always translated into funding decisions. However, given that the (non-binding) HTA recommendations in England, Scotland and Australia have been found to largely shape the final funding decisions in these countries [57], we treated the HTA outcomes across all study countries as “funding decisions”; based on that, the terms “recommendation”, “decision” and “decision outcome” all refer to “funding decisions” and have been used interchangeably throughout the text.

Finally, none of the MEAs included in this analysis were implemented across multiple indications of a specific molecule and/or were part of a MYMI agreement. As such, we acknowledge that in our impact assessment study we do not account for and/or explicitly discuss the potential benefits in patient access arising from the novel approach of applying MEAs across multiple indications and years. This approach arises as an increasingly promising strategy to achieve faster and broader patient access by reducing the administrative burden associated with conducting the same upfront evaluation process for each indication of the same product, while aligning price to the value that the product offers for each indication without the need for indication-based pricing [58]. Nevertheless, the introduction of MYMI agreements is also subject to country specific legal arrangements which can contribute to unnecessary delays in the negotiation process. Therefore, understanding the extent to which MYMI agreements can enhance the positive impact of traditional MEA mechanisms on greater and more timely access to medicines, especially in oncology, arises as a priority topic for future impact assessment studies on MEAs.

## Conclusions

Despite the application of MEAs being heterogenous across countries and often associated with high administrative burden and potential time delays, MEAs can still contribute to enhanced accessibility at the level of individual countries by allowing patient access to medicines that would not be reimbursed otherwise. However, presence of a MEA itself does not necessarily grant a timely and favourable funding decision as other factors such as the quality of clinical evidence submitted, and the type of endpoint used therein are also paramount in shaping the final funding decision and the respective timelines to decision-making. Of course, even though MEAs offer a higher likelihood for positive reimbursement, the question remains on whether the technologies approved with a MEA add true value in outcomes for patients and healthcare systems, whether they truly address the decision-making uncertainties characterising a technology and whether outcomes-based schemes measure meaningful clinical markers from the payers’ and patients’ perspective. Overall, it arises that only if applied strategically, MEAs can become a mainstay in the future of medicine availability, in reducing the financial burden for healthcare systems and in allowing faster access to new, innovative medicines.

## Supplementary Information

**Additional file 1: Appendix Table 1.** Study countries, their HTA agencies and respective perspective taken into HTA decision- making. **Appendix Table 2.** Information about all medicine-indication pairs (per country) studied in this analysis. **Appendix Table 3.** Descriptive statistics on the final funding decision outcomes after resubmission and statistical significance (p) of their HTA determinants across all sample. **Appendix Table 4.** Time (days) from initial to final funding decision after resubmission, and statistical significance (p) of their HTA determinants across all sample.

## Data Availability

The datasets used and/or analysed during the current study are available from the corresponding author on reasonable request.

## Footnotes

1 The effects of the two variables found by the *χ*^*2*^ tests to be statistically significant in determining the funding decision outcome following resubmission of evidence for a previously rejected medicine-indication pair (*i.e.,* resubmission with vs. with MEA and resubmission with vs. without cost effectiveness uncertainties) could not be studied together in the same model due to violation in the assumption of multicollinearity Therefore, their relevant effects were studied by including only one of the two variables in different models and subsequently, comparing their contribution and significance between the respective models.
