# Supplementary material for: Impact of Managed Entry Agreements on availability of and timely access to medicines: an ex-post evaluation of agreements implemented for oncology therapies in four countries
Source: BMC Health Serv Res. 2022 Aug 20;22:1066. doi: 10.1186/s12913-022-08437-w (PMC9392357; doi:10.1186/s12913-022-08437-w)
Supplement: Supplementary file 1 — Additional file 1: Appendix Table 1. Study countries, their HTA agencies and respective perspective taken into HTA decision- making. Appendix Table 2. Information about all medicine-indication pairs (per country) studied in this analysis. Appendix Table 3. Descriptive statistics on the final funding decision outcomes after resubmission and statistical significance (p) of their HTA determinants across all sample. Appendix Table 4. Time (days) from initial to final funding decision after resubmission, and statistical significance (p) of their HTA determinants across all sample. [file 12913_2022_8437_MOESM1_ESM.docx]

Appendix Table 1. Study countries, their HTA agencies and respective perspective taken into HTA decision- making.

| **Study country** | **HTA body** | **HTA perspective** | **HTA Website searched** |
| --- | --- | --- | --- |
| England | NICE: National Institute for Health and Care Excellence | Clinical and cost-effectiveness, national health and personal social services perspective | <https://www.nice.org.uk> |
| Scotland | SMC: Scottish Medicines Consortium | Clinical and cost-effectiveness, national health and personal social services perspective | <https://www.scottishmedicines.org.uk> |
| Sweden | TLV: Dental and Pharmaceutical Benefits Board | Clinical and cost-effectiveness, societal perspective | <https://www.tlv.se/in-english.html> |
| Australia | PBAC: Pharmaceutical Benefits Advisory Committee | Clinical and cost-effectiveness, national health system and societal perspective | <https://www.pbs.gov.au/info/industry/listing/elements/pbac-meetings> |
|  |  |  |  |
| ***Note:*** | The health economic evaluation takes either a societal perspective – taking into account indirect costs of treatment and illnesses (as in Sweden) – or a health system perspective, in which only direct costs to the healthcare system are considered (as in England and Scotland). Some countries employ a mix of societal and health system perspectives. | | |

**Appendix Table 2.** Information about all medicine-indication pairs (per country) studied in this analysis.

| **National Competent Authority** | **Molecule name** | **Brand name** | **Manufacturer** | **ATC classification** | **Indication under review (as per EMA)** |
| --- | --- | --- | --- | --- | --- |
| NICE | Ipilimumab-1 | Yervoy® | Bristol-Myers Squibb | L01XC11 | Treatment of advanced (unresectable or metastatic) melanoma in adults who have received prior therapy |
| NICE | Bevacizumab-1 | Avastin® | Hoffmann-La Roche | L01XC07 | In combination with carboplatin and paclitaxel for 'the front-line treatment of advanced (International Federation of Gynaecology and Obstetrics [FIGO] stages IIIB, IIIC and IV) epithelial ovarian, fallopian tube or primary peritoneal cancer |
| NICE | Eribulin-1 | Halaven® | Eisai | L01XX41 | Halaven® with the active substance eribulin is approved as a monotherapy for the treatment of patients with locally advanced or metastatic breast cancer who have progressed after at least two chemotherapies for the treatment of advanced breast cancer. The pre-treatment regimens should contain an anthracycline and a taxane, unless these treatments were not suitable for the patient. |
| NICE | Paclitaxel | Abraxane® | Celgene | L01CD01 | Abraxane® in combination with gemcitabine is indicated for the first-line treatment of adult patients with metastatic adenocarcinoma of the pancreas. |
| NICE | Pomalidomide | Imnovid® | Celgene | L04AX06 | In combination with dexamethasone, phlalidomide (Imnovid®) is indicated for the treatment of recurrent and refractory multiple myeloma in adult patients who have received at least two previous therapies, including lenalidomide and bortezomib, and have shown a progression under the last therapy. |
| NICE | Cabazitaxel | Jevtana® | Sanofi-Aventis | L01CD04 | Jevtana® in combination with prednisone or prednisolone is indicated for the treatment of patients with hormone-refractory metastatic prostate cancer previously treated with a docetaxel-containing regimen. |
| PBAC | Osimertinib | Tagrisso® | AstraZeneca | L01XE35 | In patients with non-small cell lung cancer whose cancer is advanced or has spread and has a particular mutation called T790M. The mutation is a change in the gene of the protein epidermal growth factor receptor, EGFR |
| PBAC | Idelalisib-1 | Zydelig® | Gilead | L01XX47 | In combination with rituximab for the treatment of adult patients with chronic lymphocytic leukaemia (CLL): • who have received at least one prior therapy, or • as first line treatment in the presence of 17p deletion or TP53 mutation in patients unsuitable for chemo-immunotherapy |
| PBAC | Olaparib | Lynparza® | AstraZeneca | L01XX46 | Monotherapy (alone) for maintenance therapy for ovarian cancer recurrence in patients with a specific mutation, BRCA |
| PBAC | Eribulin-1 | Halaven® | Eisai | L01XX41 | Halaven® with the active substance eribulin is approved as a monotherapy for the treatment of patients with locally advanced or metastatic breast cancer who have progressed after at least two chemotherapies for the treatment of advanced breast cancer. The pre-treatment regimens should contain an anthracycline and a taxane, unless these treatments were not suitable for the patient. |
| PBAC | Palbociclib | Ibrance® | Pfizer | L01XE33 | In patients with hormone receptor (HR) positive, human epidermal growth factor receptor 2 (HER2) negative locally advanced or metastatic breast cancer |
| PBAC | Nintedanib-2 | Vargatef® | Boehringer Ingelheim | L01XE31 | Nintedanib (Vargatef®) is used in combination with docetaxel for the treatment of adult patients with locally advanced, metastatic or locally recurrent non-small-cell lung carcinoma (NSCLC) with adenocarcinoma histology after first-line chemotherapy. |
| PBAC | Lenvatinib | Lenvima® | Eisai | L01XE29 | Lenvima® is indicated for the treatment of adult patients with progressive, locally advanced or metastatic, differentiated (papillary/follicular/Hürthle cell) thyroid carcinoma (DTC), refractory to radioactive iodine (RAI). |
| PBAC | Axitinib | Inlyta® | Pfizer | L01XE17 | Treating adults with advanced renal cell carcinoma after failure of treatment with a first‑line tyrosine kinase inhibitor or a cytokine |
| PBAC | Blinatumomab | Blincyto® | Amgen | L01XC19 | Previously treated Philadelphia-chromosome-negative acute lymphoblastic leukaemia |
| PBAC | Pembrolizumab-3 | Keytruda® | Merck Sharp & Dohme | L01XC18 | First-line treatment of metastatic NSCLC with PD-L1 expressing tumours (TPS ≥ 50%) without activating EGFR or ALK mutations in adults |
| PBAC | Nivolumab-3 | Opdivo® | Bristol-Myers Squibb | L01XC17 | Renal Cell Carcinoma (RCC); Opdivo(r) is indicated as monotherapy in adults for the treatment of advanced renal cell carcinoma after pretreatment. |
| PBAC | Nivolumab-1 | Opdivo® | Bristol-Myers Squibb | L01XC17 | Opdivo® is indicated as a monotherapy in adults for the treatment of advanced (non-resectable or metastatic) melanoma. |
| PBAC | Nivolumab-6 | Opdivo® | Bristol-Myers Squibb | L01XC17 | Opdivo® is indicated in adults for the treatment of advanced (non-resectable or metastatic) melanoma in combination with ipilimumab |
| PBAC | Obinutuzumab-1 | Gazyvaro® | Hoffmann-La Roche | L01XC15 | In combination with chlorambucil for adults with untreated chronic lymphocytic leukaemia who have comorbidities that make full‑dose fludarabine‑based therapy unsuitable for them |
| PBAC | Trastuzumab emtansine-1 | Kadcyla® | Hoffmann-La Roche | L01XC14 | Trastuzumab Emtansin (Kadcyla®) is indicated as a single agent for the treatment of adult patients with HER2-positive, inoperable locally advanced or metastatic breast cancer who previously received, individually or in combination, trastuzumab and a taxane. Patients should either have received prior treatment for locally advanced or metastatic disease, or have developed a recurrence during or within six months after adjuvant treatment. |
| PBAC | Brentuximab Vedotin-2 | Adcetris® | Takeda | L01XC12 | For the treatment of adult patients with relapse or refractory systemic large cell anaplastic lymphoma (sALCL). |
| PBAC | Brentuximab Vedotin-3 | Adcetris® | Takeda | L01XC12 | Adcetris® is used for the treatment of adult patients with CD30 + HL with increased recurrence or progressive risk after an ASCT |
| PBAC | Pazopanib-1 | Votrient® | GlaxoSmithKline | L01XE11 | Votrient® T is indicated for the treatment of advanced and/or metastatic renal cell carcinoma (RCC). |
| PBAC | Cabazitaxel | Jevtana® | Sanofi-Aventis | L01CD04 | Jevtana® in combination with prednisone or prednisolone is indicated for the treatment of patients with hormone-refractory metastatic prostate cancer previously treated with a docetaxel-containing regimen. |
| PBAC | Ribociclib | Kisqali ® | Novartis | L01XE42 | Treating hormone receptor-positive, human epidermal growth factor receptor 2‑negative, locally advanced or metastatic breast cancer as initial endocrine-based therapy in adults |
| PBAC | Nivolumab-2 | Opdivo® | Bristol-Myers Squibb | L01XC17 | In adult patients with locally advanced or metastatic non-squamous non-small cell lung cancer (NSCLC) after prior chemotherapy. |
| PBAC | Nivolumab-4 | Opdivo® | Bristol-Myers Squibb | L01XC17 | Treating squamous cell carcinoma of the head and neck in adults whose disease has progressed on platinum-based chemotherapy |
| PBAC | Everolimus-3 | Afinitor® | Novartis | L01XE10 | For treatment of post menopausal women with hormone receptor-positive advanced breast cancer in combination with exemestane, after progression or recurrence (failure) on NSAI therapy. |
| PBAC | Ruxolitinib-1 | Jakavi® | Novartis | L01XE18 | For the treatment of myelofibrosis in adults who have enlarged spleen or symptoms related to the disease, such as fever, night sweats, skeletal pain and weight loss. The drug is also used in secondary myelofibrosis |
| PBAC | Enzalutamide-2 | Xtandi® | Astellas Pharma | L02BB04 | Enzalutamide (Xtandi®) is indicated for the treatment of adult men with metastatic castration-resistant prostate carcinoma with asymptomatic or mild symptomatic course after failure of androgen withdrawal therapy, in which chemotherapy has not yet been clinically indicated. |
| PBAC | Pomalidomide | Imnovid® | Celgene | L04AX06 | In combination with dexamethasone, phlalidomide (Imnovid®) is indicated for the treatment of recurrent and refractory multiple myeloma in adult patients who have received at least two previous therapies, including lenalidomide and bortezomib, and have shown a progression under the last therapy. |
| PBAC | Ponatinib | Iclusig® | ARIAD pharmaceuticals | L01XE24 | For the treatment of two types of blood cancer, chronic myeloid leukemia (KML) and Philadelphia chromosomal acute lymphocytic leukemia (Ph + ALL) |
| PBAC | Dabrafenib-1 | Tafinlar® | GlaxoSmithKline | L01XE23 | As monotherapy for the treatment of adult patients with unresectable or metastatic melanoma with a BRAF V600 mutation. |
| PBAC | Crizotinib-1 | Xalkori® | Pfizer | L01XE16 | Treatment of advanced non-small cell lung cancer (NSCLC) with positive anaplastic lymphoma kynase (ALK+) for adult patients previously treated with at least one other lung cancer treatment |
| PBAC | Ipilimumab-1 | Yervoy® | Bristol-Myers Squibb | L01XC11 | Treatment of advanced (unresectable or metastatic) melanoma in adults who have received prior therapy |
| PBAC | Brentuximab Vedotin-1 | Adcetris® | Takeda | L01XC12 | Adcetris ® is indicated for the treatment of adult patients with relapsed or refractory CD30+ Hodgkin lymphoma (HL):following autologous stem cell transplant (ASCT) or following at least two prior therapies when ASCT or multi-agent chemotherapy is not a treatment option. |
| PBAC | Ibrutinib-1 | Imbruvica® | Janssen-Cilag | L01XE27 | Previously treated chronic lymphocytic leukaemia and untreated chronic lymphocytic leukaemia with 17p deletion or TP53 mutation |
| PBAC | Idelalisib-2 | Zydelig® | Gilead | L01XX47 | Treatment of relapsed/refractory follicular lymphoma (FL) that has progressed despite prior treatment with rituximab and an alkylating agent. |
| SMC | Daratumumab | Darzalex® | Janssen-Cilag | L01XC24 | Darzalex® is indicated as a monotherapy for the treatment of adult patients with recurrent and refractory multiple myeloma who have already been treated with a proteasome inhibitor and an immune modulator and have shown a disease progression during the last therapy. |
| SMC | Olaparib | Lynparza® | AstraZeneca | L01XX46 | Monotherapy (alone) for maintenance therapy for ovarian cancer recurrence in patients with a specific mutation, BRCA |
| SMC | Crizotinib-1 | Xalkori® | Pfizer | L01XE16 | Treatment of advanced non-small cell lung cancer (NSCLC) with positive anaplastic lymphoma kynase (ALK+) for adult patients previously treated with at least one other lung cancer treatment |
| SMC | Nivolumab-3 | Opdivo® | Bristol-Myers Squibb | L01XC17 | Renal Cell Carcinoma (RCC): Opdivo® is indicated as monotherapy in adults for the treatment of advanced renal cell carcinoma after pretreatment. |
| SMC | Nivolumab-1 | Opdivo® | Bristol-Myers Squibb | L01XC17 | Opdivo® is indicated as a monotherapy in adults for the treatment of advanced (non-resectable or metastatic) melanoma. |
| SMC | Paclitaxel | Abraxane® | Celgene | L01CD01 | Abraxane® in combination with gemcitabine is indicated for the first-line treatment of adult patients with metastatic adenocarcinoma of the pancreas. |
| SMC | Cabazitaxel | Jevtana® | Sanofi-Aventis | L01CD04 | Jevtana® in combination with prednisone or prednisolone is indicated for the treatment of patients with hormone-refractory metastatic prostate cancer previously treated with a docetaxel-containing regimen. |
| SMC | Ipilimumab-1 | Yervoy® | Bristol-Myers Squibb | L01XC11 | Treatment of advanced (unresectable or metastatic) melanoma in adults who have received prior therapy |
| SMC | Pertuzumab-2 | Perjeta® | Hoffmann-La Roche | L01XC13 | Perjeta®, in combination with trastuzumab and chemotherapy in adult patients, is indicated for the neoadjuvant treatment of HER2-positive locally advanced, inflammatory or early breast cancer with high recurrence risk |
| SMC | Everolimus-3 | Afinitor® | Novartis | L01XE10 | For treatment of post menopausal women with hormone receptor-positive advanced breast cancer in combination with exemestane, after progression or recurrence (failure) on NSAI therapy. |
| SMC | Bosutinib | Bosulif® | Pfizer | L01XE14 | Treatment of adult patients with chronic phase (CP), accelerated phase (AP), and blast phase (BP) Philadelphia chromosome positive chronic myelogenous leukaemia (Ph+ CML) previously treated with one or more tyrosine kinase inhibitor(s) and for whom imatinib, nilotinib and dasatinib are not considered appropriate treatment options |
| SMC | Vemurafenib | Zelboraf® | Hoffmann-La Roche | L01XE15 | Vemurafenib is indicated in monotherapy for the treatment of adult patients with BRAF-V600-mutation-positive unresectable or metastatic melanoma |
| SMC | Aflibercept-5 | Zaltrap® | Sanofi-Aventis | L01XX44 | Zaltrap® in combination with chemotherapy consisting of irinotecan / 5-fluorouracil / folinic acid (FOLFIRI) is used in adults with metastatic colorectal carcinoma (MCRC) who have undergone under or after an oxaliplatin-containing regimen. |
| SMC | Abiraterone Acetate-1 | Zytiga® | Janssen-Cilag | L02BX03 | In combination with prednisone or prednisolone for the treatment of metastatic castration-resistant prostate carcinoma in adult men whose disease is progressive during or after docetaxel-containing chemotherapy. |
| SMC | Pomalidomide | Imnovid® | Celgene | L04AX06 | In combination with dexamethasone, phlalidomide (Imnovid®) is indicated for the treatment of recurrent and refractory multiple myeloma in adult patients who have received at least two previous therapies, including lenalidomide and bortezomib, and have shown a progression under the last therapy. |
| TLV | Vismodegib | Erivedge® | Hoffmann-La Roche | L01XX43 | Erivedge® is indicated for the treatment of adult patients with: - symptomatic metastatic basal cell carcinoma - locally advanced basal cell carcinoma inappropriate for surgery or radiotherapy |
| TLV | Vemurafenib | Zelboraf® | Hoffmann-La Roche | L01XE15 | Vemurafenib is indicated in monotherapy for the treatment of adult patients with BRAF-V600-mutation-positive unresectable or metastatic melanoma |
| TLV | Cabozantinib-1 | Cabometyx® | Ipsen ltd. | L01XE26 | Treatment of advanced renal cell carcinoma (RCC) in adults following prior vascular endothelial growth factor (VEGF)-targeted therapy |
| TLV | Enzalutamide-1 | Xtandi® | Astellas Pharma | L02BB04 | Enzalutamide (Xtandi®) is indicated for the treatment of adult men with metastatic castration-resistant prostate cancer whose disease progresses during or after chemotherapy with docetaxel. |
| TLV | Enzalutamide-2 | Xtandi® | Astellas Pharma | L02BB04 | Enzalutamide (Xtandi®) is indicated for the treatment of adult men with metastatic castration-resistant prostate carcinoma with asymptomatic or mild symptomatic course after failure of androgen withdrawal therapy, in which chemotherapy has not yet been clinically indicated. |

Appendix Table 3. Descriptive statistics on the final funding decision outcomes after resubmission and statistical significance (p) of their HTA determinants across all sample.

| **Funding decision outcome after resubmission, across all sample (n=59)** | | | | | | |
| --- | --- | --- | --- | --- | --- | --- |
| List (L) | | 1 (1.7%) | | | | |
| List with restrictions | LWC | 5 (8.5%) | | | | |
|  | LWCMEA | 45 (76.3%) | | | | |
| Do not List (DNL) | | 8 (13.5%) | | | | |
| **Funding decisions following resubmissions per country** | | | | | | |
|  | | **Remained non-favourable (DNL)** | **Reversed to favourable (L/LWC/LWCMEA)** | | | ***p*-value** |
|  |  |  | **L** | **LWC** | **LWCMEA** |  |
| England (NICE) (n=6) | | 0% | 0% | 1 (16.7%) | 5 (83.3%) | *.163* |
| Australia (PBAC) (n=33) | | 7 (21%) | 0% | 4 (12%) | 22 (67%) |  |
| Scotland (SMC) (n=15) | | 0% | 1 (6.7%) | 0% | 14 (93.3%) |  |
| Sweden (TLV) (n=5) | | 1 (20%) | 0% | 0% | 4 (80%) |  |
| **HTA determinants of funding decision following resubmission** | | | | | | |
|  | | **Non- favourable (DNL)**  **(n=8)** | **Favourable**  **(L/LWC/LWCMEA)**  **(n=51)** | | | ***p*-value** |
| **Molecule specific characteristics** | | | | | | |
| **MEA in place** | | | | | | |
| Yes | | 2 (25%) | 45 (88%) | | | ***< .000*** |
| No | | 6 (75%) | 6 (12%) | | |  |
| **Endpoint** | | | | | | |
| Surrogate | | 5 (62.5%) | 27 (53%) | | | .342 |
| Clinical | | 1(12.5%) | 18 (35%) | | |  |
| Combination | | 2 (25%) | 5 (10%) | | |  |
| n/a* | | 0% | 1 (2%) | | |  |
| **Rarity** | | | | | | |
| Orphan | | 1 (12.5%) | 15 (29.4%) | | | .317 |
| Non orphan | | 7 (87.5%) | 36 (70.6%) | | |  |
| **Type of MA** | | | | | | |
| Standard | | 6 (25%) | 37 (72.5%) | | | .885 |
| Non-standard | | 2 (75%) | 14 (27.5%) | | |  |
| **Study type** | | | | | | |
| RCT | | 7 (87.5%) | 48 (94%) | | | .489 |
| Non-RCT /Observational | | 1 (12.5%) | 3 (6%) | | |  |
| **Social value judgments** | | | | | | |
| **Disease severity** | | | | | | |
| Considered | | 2 (25%) | 15 (29.4%) | | | *.798* |
| Not considered | | 6 (75%) | 36 (70.6%) | | |  |
| **Unmet need** | | | | | | |
| Considered | | 4 (50%) | 31(60.8%) | | | *.564* |
| Not considered | | 4 (50%) | 20 (39.2%) | | |  |
| **Administration advantage** | | | | | | |
| Considered | | 0% | 16 (31.4%) | | | *.063* |
| Not considered | | 8 (100%) | 35 (68.6%) | | |  |
| **Innovation** | | | | | | |
| Considered | | 1 (12.5%) | 18 (35.3%) | | | *.20* |
| Not considered | | 7 (87.5%) | 33 (64.7%) | | |  |
| **Short life expectancy** | | | | | | |
| Considered | | 0% | 8 (15.7%) | | | *.228* |
| Not considered | | 8 (100%) | 43 (84.3%) | | |  |
| **Societal impact** | | | | | | |
| Considered | | 0% | 7 (13.7%) | | | .264 |
| Not considered | | 8 (100%) | 44 (86.3%) | | |  |
| **Special Considerations (i.e., end-of-life criteria)** | | | | | | |
| Considered | | 2 (25%) | 17 (33.3%) | | | .639 |
| Not considered | | 6 (75%) | 34 (66.6%) | | |  |
| **Clinical uncertainties** | | | | | | |
| **Clinical benefit** | | | | | | |
| Raised | | 7 (87.5%) | 32 (62.7%) | | | *.169* |
| Not raised | | 1 (12.5%) | 19 (37.3%) | | |  |
| **Study design** | | | | | | |
| Raised | | 2 (25%) | 18 (35.3%) | | | *.567* |
| Not raised | | 6 (75%) | 33 (64.7%) | | |  |
| **Relevance to clinical practice** | | | | | | |
| Raised | | 1(12.5%) | 12 (32.5%) | | | *.484* |
| Not raised | | 7(87.5%) | 39 (76.5%) | | |  |
| **Population generalizability** | | | | | | |
| Raised | | 1(12.5%) | 8 (15.7%) | | | *.816* |
| Not raised | | 7(87.5%) | 43 (84.3%) | | |  |
| **Clinical comparator** | | | | | | |
| Raised | | 2 (25%) | 17 (33.3%) | | | *.639* |
| Not raised | | 6 (75%) | 34 (66.6%) | | |  |
| **Clinical evidence** | | | | | | |
| Raised | | 4 (50%) | 22 (%) | | | *.716* |
| Not raised | | 4 (50%) | 29 (%) | | |  |
| **Economic uncertainties** | | | | | | |
| **Cost effectiveness** | | | | | | |
| Raised | | 6 (75%) | 27 (52.3%) | | | ***.027*** |
| Not raised | | 2 (25%) | 24 (47%) | | |  |
| **Utilities** | | | | | | |
| Raised | | 3 (37.5%) | 10 (19.6%) | | | *.256* |
| Not raised | | 5 (62.5%) | 41 (80.4%) | | |  |
| **Costs** | | | | | | |
| Raised | | 6 (75%) | 24 (47%) | | | *.142* |
| Not raised | | 2 (25%) | 27 (53%) | | |  |
| **Modelling** | | | | | | |
| Raised | | 6 (75%) | 30 (58.8%) | | | *.383* |
| Not raised | | 2 (25%) | 21 (41.2%) | | |  |
| **Model type** | | | | | | |
| Raised | | 0%) | 1 (2%) | | | *.690* |
| Not raised | | 8 (100%) | 50 (98%) | | |  |
| **Economic comparator** | | | | | | |
| Raised | | 0%) | 5 (10%) | | | *.355* |
| Not raised | | 8 (100%) | 46 (90%) | | |  |
|  | |  |  | | |  |
| ***Key:*** | | *n/a: endpoint not applicable for the type of study used in the evidence submitted (i.e., indirect comparison***,*** health economic report). | | | | |
| ***Note:*** | | - L: List, LWC: List with criteria; LWCMEA: List with criteria which include a MEA; DNL: Do not list. - HTA: Heath Technology Assessment, MA: Marketing authorization; MEA: Managed Entry Agreement, RCT: Randomized Controlled Trial. - PBAC: Pharmaceutical Benefits Advisory Committee, NICE: National Institute for Health and Care Excellence, SMC: Scottish Medicines Consortium, TLV: Dental and Pharmaceutical Benefits Board. | | | | |

Appendix Table 4. Time (days) from initial to final funding decision after resubmission, and statistical significance (*p*) of their HTA determinants across all sample.

| **Time from previous submission to final funding decision** | | | |
| --- | --- | --- | --- |
|  | **n (%)** | **Days, mean (SD)** | |
| Resubmission with MEA | 47 (66%) | 452 (±364) | |
| Resubmission without MEA | 12 (17%) | 404 (±254) | |
| Resubmission after MEA expiry | 12 (17%) | 935 (±330) | |
| **Time determinants** | | | |
|  | **Days, mean (SD)** | | ***p*- value** |
| **HTA agency** | | | |
| England (NICE) | 938 (±559) | | ***.000*** |
| Australia (PBAC) | 378 (±242) | |  |
| Scotland (SMC) | 342 (±249) | |  |
| Sweden (TLV) | 837 (±302) | |  |
|  | **Molecule specific characteristics** | |  |
|  | **MEA in place** | |  |
| Yes | 550 (±404) | | *.394* |
| No | 404 (±254) | |  |
|  | **MEA Type** | |  |
| Financial | 476 (±407) | | ***.002*** |
| Outcomes-based | 957 (±231) | |  |
| Combination | 422 (±231) | |  |
|  | **Endpoint** | |  |
| Surrogate | 514 (±324) | | *.659* |
| Clinical | 494 (±514) | |  |
| Surrogate & Clinical | 570 (±351) | |  |
| n/a* | 380 (±0) | |  |
|  | **Rarity** | |  |
| Orphan | 554 (±372) | | *.559* |
| Non orphan | 512 (±396) | |  |
|  | **Type of MA** | |  |
| Standard | 491 (±397) | | *.182* |
| Non-standard | 597 (±359) | |  |
| **Social value judgments** | | | |
|  | **Disease severity** | |  |
| Considered | 687 (±432) | | ***.013*** |
| Not considered | 437 **(**±335) | |  |
| **Unmet need** | | | |
| Considered | 504 (±414) | | *.465* |
| Not considered | 539 (±349) | |  |
| **Administration advantage** | | | |
| Considered | 487 (±472) | | *.370* |
| Not considered | 529 (±357) | |  |
| **Innovation** | | | |
| Considered | 492 (±454) | | *.397* |
| Not considered | 530 (±356) | |  |
| **Short life expectancy** | | | |
| Considered | 719 (±598) | | *.290* |
| Not considered | 486 (±333) | |  |
| **Societal impact** | | | |
| Considered | 282 (±182) | | ***.044*** |
| Not considered | 554 (±395) | |  |
| **Special Considerations (i.e., end-of-life criteria)** | | | |
| Considered | 618 (±462) | | .179 |
| Not considered | 467 (±332) | |  |
| **Clinical uncertainties** | | | |
| **Clinical benefit** | | | |
| Raised | 528 (±385) | | *.718* |
| Not raised | 500 (±394) | |  |
| **Study design** | | | |
| Raised | 574 (±472) | | .726 |
| Not raised | 492 (±337) | |  |
| **Relevance to clinical practice** | | | |
| Raised | 624 (±379) | | .150 |
| Not raised | 480 (±384) | |  |
| **Population generalizability** | | | |
| Raised | 545 (±596) | | *.233* |
| Not raised | 514 (±332) | |  |
| **Clinical comparator** | | | |
| Raised | 563 (±471) | | .918 |
| Not raised | 500 (±345) | |  |
| **Clinical evidence** | | | |
| Raised | 471 (±353) | | *.284* |
| Not raised | 561 (±411) | |  |
| **Economic uncertainties** | | | |
| **Cost effectiveness** | | | |
| Raised | 583 (±433) | | *.304* |
| Not raised | 429 (±283) | |  |
| **Utilities** | | | |
| Raised | 572 (±529) | | *.889* |
| Not raised | 503 (±336) | |  |
| **Costs** | | | |
| Raised | 497 (±405) | | .458 |
| Not raised | 539 (±370) | |  |
| **Modelling** | | | |
| Raised | 491 (±389) | | *.311* |
| Not raised | 563 (±381) | |  |
| **Model type** | | | |
| Raised | 365 (±0) | | .785 |
| Not raised | 521 (±387) | |  |
| **Economic comparator** | | | |
| Raised | 727 (±420) | | *.099* |
| Not raised | 499 (±379) | |  |
|  |  | |  |
| ***Key:***  *n/a: endpoint not applicable for the type of study used in the evidence submitted (i.e., indirect comparison). | | | |
| ***Note:***  HTA: Heath Technology Assessment, MA: Marketing authorization; MEA: Managed Entry Agreement, SD: Standard deviation. PBAC: Pharmaceutical Benefits Advisory Committee, NICE: National Institute for Health and Care Excellence, SMC: Scottish Medicines Consortium, TLV: Dental and Pharmaceutical Benefits Board. | | | |
